# A Comparison of Tip-Apex Distance at Various Angles of Fixation Devices in Hip Fractures

**DOI:** 10.7759/cureus.35576

**Published:** 2023-02-28

**Authors:** Mohammed Ali, Oday Al-Dadah

**Affiliations:** 1 Trauma and Orthopaedics, South Tyneside District Hospital, South Shields, GBR; 2 Trauma and Orthopaedics, South Tyneside and Sunderland National Health Service (NHS) Foundation Trust, South Shields, GBR

**Keywords:** neck of the femur fracture, tip-apex distance, fixed-angle fixation, intramedullary nail, dynamic hip screw

## Abstract

Background

Intertrochanteric neck of the femur (NOF) fractures are very common, and the majority are fixed using dynamic hip screws (DHS) or intramedullary (IM) nails with a fixed angle. The aim of this study was to assess which angle of fixation has a better tip-apex distance (TAD) on X-ray and lower complication rates.

Methods

We included patients with intertrochanteric hip fractures fixed using a DHS or an IM nail. We included patients who had complete radiological and clinical records and a minimum follow-up of 24 months. We measured the TAD and recorded the number of implant cutouts, fracture site nonunions, and periprosthetic fractures.

Results

A total of 107 patients were included, 35 IM nails and 72 DHS. There were four cases of implant cutouts within the DHS group and none in the IM nail group. All four cutout cases were fixed using 135° angle DHS, and two had TAD of more than 25 mm. Multivariable regression analysis revealed that the implant fixation device (p=0.002) and the angle of fixation (p<0.001) are the most important predictors of TAD.

Conclusion

Smaller angle (130° or 125°) fixation devices allow better positioning of the lag screw and consequently better TAD, which leads to a lower probability of implant cutout in patients undergoing fracture of the neck of the femur surgery.

## Introduction

Neck of the femur (NOF) fracture in the geriatric population is one of the most frequent presentations in hospitals in the United Kingdom (UK) [1]. The estimated number of admissions is about 80,000 annually, and the annual cost implications are nearly two billion pounds. It is one of the commonest causes of fracture-related death in the United Kingdom, and the incidence is expected to reach a hundred thousand by 2030 [1]. Intertrochanteric neck of the femur (NOF) fractures are prevalent, and the majority are fixed using a dynamic hip screw (DHS) or, occasionally, an intramedullary (IM) nail with a fixed angle. As an entity, extracapsular neck of the femur fractures are almost twice as common as intracapsular fractures. According to the National Hip Fracture Database report for December 2021, 62% of intertrochanteric fractures were fixed using DHS [2]. The fixation failure rate has been reported to be between 5% and 15% [3,4]. When the fracture collapses into a varus, the screw can cut out through the femoral head, eventually leading to a mechanical failure. The risk of cutouts is higher in elderly or osteoporotic patients and those with unstable fractures. This risk is further increased in the presence of poor fracture site reduction and poor implant fixation [4-9]. Therefore, it is crucial to place the lag screw in the femoral head accurately.

Baumgaertner et al. [10] in 1995 described the concept of the tip-apex distance (TAD). Their study concluded that a TAD greater than 25 mm has a higher risk of cutout and mechanical failure. Andruszkow et al. [9] conducted a retrospective study of 235 patients with extracapsular fractures treated with a DHS or an intramedullary nail. A TAD of more than 25 mm was shown to be an associated factor with cutouts in stable and unstable fractures. Different angles are manufactured for the DHS and IM nail and vary from 125° to 150°. The device angle can be determined by measuring the contralateral side neck-shaft angle; however, this is not always reliable. When using a 135° angled implant, it is noted that the lag screw was often placed inferiorly within the femoral neck to achieve the screw tip centralized in the femoral head and attain a TAD of less than 25 mm. Additionally, 135° angled implants are noted to be more suitable in cases with valgus neck-shaft angles. Andruszkow et al. [9] highlighted that fracture reduction with a valgus neck-shaft angle of 5°-10° was associated with a trend toward a lower rate of screw cutout; however, this is not achievable in all fractures.

The aim of this study was to compare the TAD and complication rates of various angled implants in NOF fracture fixation surgery.

## Materials and methods

This was a retrospective analysis of patients’ data; hence, institutional review board (IRB)/ethics committee approval was not required. Data was collected from the trauma theater records, patient notes, outpatient clinic letters, and the Picture Archiving and Communication System (PACS) (Centricity version 6, GE Healthcare, Chicago, USA) for X-ray evaluation. We studied all patients with NOF fractures treated with a fixed-angle device, including DHS and IM nails. We included patients with complete radiological and clinical records and a minimum follow-up of 24 months. All surgeries were performed by experienced surgeons who know the TAD concept. Operating surgeons used similar image intensifier machines with similar spatial resolution, gain, and pixel size. All surgical implants were available, and surgeons were not limited to a particular implant. The patient’s age, gender, and fracture patterns were recorded. All complications during the two years postoperative period were also recorded. The setscrews of the IM nails were not tightened in simple intertrochanteric fractures as this will stop impaction. For reverse-oblique and subtrochanteric fractures, as no sliding occurred between the head-neck and the shaft, there is no difference in whether the setscrew is tightened or not tightened.

**Figure 1:**
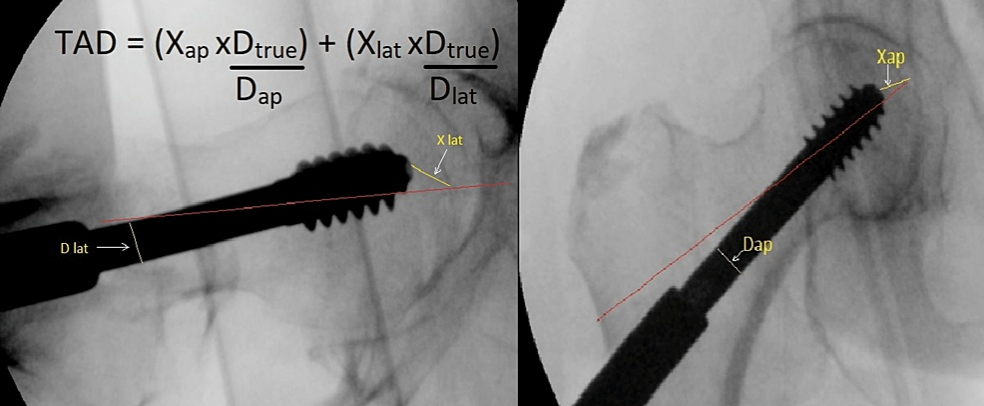
Measurement of the Tip-Apex Distance Illustration of the TAD with its corresponding equation for correcting radiographic magnification TAD: tip-apex distance, Xap and Xlat: the measured distance on the anteroposterior and lateral views, Dtrue: the actual diameter of the lag screw, Dap and Dlat: the measured diameter of the lag screw on the anteroposterior and lateral views

Baumgaertner et al. [10] categorized reduction as good, acceptable, or poor. A good reduction had a normal or mildly valgus neck-shaft angle on the anteroposterior (AP) view, less than 20° of angulation on the lateral view, and displacement of less than 4 mm on both views. Acceptable reductions met the requirements as regards alignment or displacement, but not both. We classified reductions as poor when they did not meet these criteria. In light of this classification, we assessed the intraoperative radiographs, and we only included good or acceptable reductions achieved prior to fixation and excluded poorly reduced fractures as they are known to have higher cutout rates and were considered a confounding factor that could bias the results of this study. The method of measuring the TAD was described by Baumgaertner et al. [10]. We used the PACS system to measure the screw diameter and the distance on both AP and lateral views (Figure 1).

Statistical analysis

Plotted histograms with fitted curve lines, box plots, normal Q-Q plots, and the Kolmogorov-Smirnov statistic were used to confirm that a normal distribution was an appropriate assumption for all continuous variables in the study. Independent-sample Student’s t-test and the one-way between-group analysis of variance (ANOVA) with Tukey’s honestly significant difference (HSD) post hoc pairwise comparisons test were used for the relevant between-group statistical analyses. Multivariable linear regression analysis was used to analyze both continuous and categorical independent variables separately to predict the dependent variable TAD. Between-group comparisons of categorical variables were analyzed using the chi-square test for independence. The level of statistical significance was set at p<0.05. Statistical analysis was performed using Statistical Package for the Social Sciences (SPSS) for Windows version 26.0 (IBM SPSS Statistics, Armonk, NY, USA).

## Results

A total of 107 patients met the inclusion criteria. These include 35 IM nails and 72 DHS. Table 1 shows the demographics of all the patients included in the study (Table 1).

**Table 1:** Demographics of the Subjects SD: standard deviation, IM nail: intramedullary nail implant fixation, DHS: dynamic hip screw implant fixation

	125° group (n=19)	130° group (n=29)	135° group (n=59)
Mean age (years) (SD)	80.5 (10.9)	81.8 (10.7)	81.5 (11.9)
Gender (male:female)	3:16	10:19	14:45
Laterality (right:left)	5:14	16:13	24:35
IM mail:DHS	19:0	10:19	6:53
Fracture type (number (%))			
Intertrochanteric	4 (21.1%)	19 (65.5%)	51 (86.4%)
Subtrochanteric	15 (78.9%)	5 (17.2%)	3 (5.1%)
Sub-capital	0 (0%)	5 (17.2%)	4 (6.8%)
Prophylactic	0 (0%)	0 (0%)	1 (1.7%)

We measured the TAD and recorded the complication rate with a particular focus on the number of lag screw cutouts, fracture site nonunions, and periprosthetic fractures due to mechanical failure. Table 2 demonstrates that 135° angled implants had the greatest number of patients with a TAD greater than 25 mm and also the greatest number of complications overall.

**Table 2:** Between-Group Complication Comparison TAD: tip-apex distance, X^2^: Pearson chi-square, 1: chi-square test for independence

	125° group (n=19)	130° group (n=29)	135° group (n=59)		
TAD (mm) (number (%))					
<25	19 (100%)	26 (89.7%)	51 (86.4%)	X^2^	2.9
>25	0 (0%)	3 (10.3%)	8 (13.6%)	p-value^1^	0.239
				Cramer’s V	0.16
Complication (number (%))					
	19 (100%)	29 (100%)	53 (89.8%)	X^2^	5.2
	0 (0%)	0 (0%)	4 (6.8%)	p-value^1^	0.522
	0 (0%)	0 (0%)	1 (1.7%)	Cramer’s V	0.16
	0 (0%)	0 (0%)	1 (1.7%)		

All the reported complications occurred in the 135° group, including four cases of lag screw cutouts, which have been further detailed in Table 3. They were predominantly female patients with intertrochanteric NOF fracture patterns treated with a DHS, but not all had a TAD greater than 25 mm (Table 3).

**Table 3:** Details of the Cutout Cases DHS: dynamic hip screw, TAD: tip-apex distance

	Case 1	Case 2	Case 3	Case 4
Age (years)	91	78	76	59
Gender	Female	Female	Male	Female
Laterality	Left	Left	Left	Right
Fracture type	Intertrochanteric	Intertrochanteric	Intertrochanteric	Intertrochanteric
Angle of implant	135°	135°	135°	135°
Implant fixation	DHS	DHS	DHS	DHS
TAD (mm)	32	35	11	22

The between-group demographic analysis shows no significant findings (Table 4), while that of the between-group angle of implant analysis showed a significantly higher TAD in the 135° group as compared to the other two groups (Table 5).

**Table 4:** Between-Group Demographic TAD Analysis 1: independent-sample Student’s t-test, TAD: tip-apex distance, IM nail: intramedullary nail implant fixation, DHS: dynamic hip screw implant fixation, SD: standard deviation, CI: confidence interval

	Gender			Laterality			Implant fixation		
	Male mean (SD)	Female mean (SD)	p-value^1^ (95% CI)	Right mean (SD)	Left mean (SD)	p-value^1^ (95% CI)	IM nail mean (SD)	DHS mean (SD)	p-value^1^ (95% CI)
TAD	16.5 (6.5)	16.7 (6.8)	0.922 (-2.8-3.1)	16.2 (6.2)	17.0 (7.1)	0.575 (-1.9-3.4)	16.1 (7.3)	17.0 (6.4)	0.549 (-1.9-3.6)

**Table 5:** Between-Group TAD Analysis TAD: tip-apex distance, 1: one-way between-group analysis of variance, 2: Tukey’s honestly significant difference post hoc pairwise comparisons, *: statistically significant at <0.05 level, SD: standard deviation, CI: confidence interval

Group (angle of implant)	TAD (mm) (mean (SD))	F statistic	p-value^1^	Eta squared	125° versus 130° mean difference (mm), p-value^2^ (95% CI)	125° versus 135° mean difference (mm), p-value^2^ (95% CI)		130° versus 135° mean difference (mm), p-value^2^ (95% CI)
125°	12.2 (4.4)	9.7	<0.001*	0.16	2.7, 0.302 (-1.6-7.1)	6.7, <0.001* (2.8-10.6)	3.9, 0.018* (0.6-7.3)	
130°	15.0 (7.2)							
135°	18.9 (6.2)							

Both the TAD-AP and the TAD-lateral are used to calculate the TAD (Table 6). However, the TAD-AP had a higher beta (b) coefficient (0.59) than TAD-lateral, which means that this independent variable (TAD-AP) makes the strongest unique contribution to explaining the dependent variable (TAD) when the variance explained by all other variables in the model is controlled for. The beta value for TAD-lateral was slightly lower (0.46), indicating that it made less of a unique contribution but still a statistically significant unique contribution to the equation, nonetheless. The age variable was also significant.

**Table 6:** Multivariable Linear Regression Analysis of TAD (Continuous Variables) TAD: tip-apex distance, AP: anteroposterior, σ_E_: root mean squared error, *: statistically significant at <0.05 level, CI: confidence interval

	Coefficient (b)	p-value	95% CI	Part correlation	R square, p-value	σ_E_
TAD-AP	0.59	<0.001*	0.98-1.11	0.38	0.98, <0.001*	0.85
TAD-lateral	0.46	<0.001*	0.78-0.92	0.29		
Age	0.03	0.010*	0.01-0.03	0.03		

Table 7 shows that gender, laterality, and fracture type are not significant predictors of TAD. However, the implant fixation device (i.e., DHS versus IM nail) and, even more so, the angle of the implant make strong contributions to explaining (predicting) the dependent variable (TAD) (Table 7).

**Table 7:** Multivariable Linear Regression Analysis of TAD (Categorical Variables) TAD: tip-apex distance, σ_E_: root mean square error, *: statistically significant at <0.05 level, CI: confidence interval

	Coefficient (b)	p-value	95% CI	Part correlation	R square, p-value	σ_E_
Gender	-0.02	0.848	-2.41-2.93	-0.02	0.24, <0.001*	6.01
Laterality	-0.01	0.926	-2.32-2.56	-0.01		
Fracture type	-0.06	0.562	-1.31-2.39	-0.05		
Implant fixation	0.39	0.002*	2.12-9.12	0.28		
Angle of implant	0.65	<0.001*	3.56-7.67	0.47		

## Discussion

The main focus of this paper is the effect of using smaller angle fixation devices on achieving better TAD. This study demonstrated that using smaller angle (130° or 125°) fixation devices allows better positioning of the lag screw and consequently better TAD, which leads to a lower probability of implant cutout in patients undergoing fracture of the neck of the femur surgery.

The factors that affect the mechanical stability of the fixation of NOF fractures were highlighted by Kaufer [11] in 1980. These include bone quality, fragment geometry, reduction, implant selection, and implant placement. The lack of one or more of these essential factors can precipitate fixation failure. The most commonly reported mode of implant failure is the penetration of the femoral head superiorly due to lag screw migration, which is known as “cutout” [12]. The well-known concept of the sliding hip screw was first popularized by Schumpelick et al. [13] in 1955 as a fixed-angle device with variable length. The sliding barrel is designed to provide compression through a tension band mechanism and transmit the forces through the medial cortex of the proximal femur [14]. Controlled fracture impaction and union are more reliably achieved when the lag screw is positioned in the subchondral bone, and progressive load sharing with the bone is maintained; this is usually accomplished when the lag screw is perpendicular to the fracture line of the femoral neck [15,16]. Although this technique would cause a medialization of the femur and reduce the femoral neck length, it has been shown to have a lower incidence of screw cutout and joint penetration when compared with earlier fixed nail/plate designs [17].

The seminal paper by Baumgaertner et al. [10] in 1995 introduced the concept of TAD and highlighted its essential role in predicting failures and screw cutouts. The measurement of screw placement does not consider the specific screw position in the femoral head. Still, it does give an accurate approximation that a deep, centrally placed screw will have a low TAD with meager chances of failure. Recently, Hsueh et al. [17] assessed 937 DHS fixations to determine the risk factors of the lag screw cutout. They found TAD to be the most predictive factor for the cutout, followed by screw position, fracture geometry, fracture reduction, and patient age.

The data analysis of the present study showed that gender, laterality, and fracture type are not significant predictors of TAD (Table 7). However, the implant fixation device (i.e., DHS versus IM nail) and, even more so, the angle of the implant make substantial contributions to explaining (predicting) the dependent variable (TAD). Although both TAD-AP and TAD-lateral are used to calculate TAD, the present study found a higher beta (b) coefficient for the former variable. The clinical implication of this is that the TAD-AP seems to influence TAD more, so it is essential not to locate/insert the lag screw too high on the AP plane, even more so than inserting the lag screw too anterior on the lateral plane (although both variables are still important overall). Age also seems to influence TAD significantly but is not as strong as the above two variables.

The present study confirmed that using a lower angle (125° or 130°) implant allows for better positioning of the lag screw and, consequently, better TAD and less chance of cutout. Radic et al. [14] suggested that a 130° angle more closely matches “normal” proximal femoral anatomy and would allow for a more straightforward trajectory for screw placement to achieve the desired TAD of 25 mm or less. They compared 130° DHS plates versus 135° DHS plates, and their study showed a statistically significant difference in TAD between them. They also found that none of the 130° plates failed in their case series, and specifically, none of the 130° plates resulted in screw cutout despite a trend toward more unstable fracture patterns in the 130° group than the 135° group, however with fewer failures and a lower TAD. A 135° plate might preferentially be used when there is a degree of coxa valga. Nayak et al. [18] assessed the results of valgus osteotomy in femoral neck fracture fixation. They reported a series of 20 patients with a fractured neck of the femur with associated high Pauwels angle, presenting late, wherein valgus osteotomy was added to their reduction and fixation and was secured with a 135° DHS. The femoral neck fractures united in 16 (80%) patients. Excellent to good results (Harris hip score > 80) were seen in 70% of the cases. The correction of the preoperative Pauwels angle changed from 68.3° to 34.3°. They concluded that the 135° DHS provides rigid internal fixation after valgus osteotomy.

When comparing IM nail and DHS, IM nail has many advantages, including a lower incidence of screw cutout and functions as a load-bearing construct in the proximal femur, predominantly through the calcar femoral [19]. Conversely, the laterally placed plate of a DHS results in an increased lever arm, thereby increasing the risk of the implant cutting out. Furthermore, biomechanically, compared to a laterally placed plate, an intramedullary positioned device (with a consequent shorter lever arm) reduces the bending force of the hip joint on the implant by 25%-30% [20]. The lateral wall of the femur in patients treated with a DHS provides a lateral buttress for controlled fracture impaction and preventing collapse. This lateral wall is fragile in unstable 31-A2 type fracture [20]. Palm et al. [21] found an eight times higher risk of reoperation due to technical failure with the gold standard technique of DHS in patients with fractures of the lateral femoral wall. This has been attributed to the fact that when the lateral femoral wall is fractured, the fracture line is parallel to the sliding vector of the sliding hip screw, which, as in the reverse oblique intertrochanteric fracture, allows the trochanteric and femoral head and neck fragments to slide laterally and the femoral shaft to slide medially. The fracture complex subsequently disintegrates, with a high risk of failure, including a cutout of the screw into the hip joint.

It is not uncommon for fractures of the lateral femoral wall to occur intraoperatively, even with standard surgical techniques, when the large-diameter hole (to accommodate the lag screw) is drilled into the lateral femoral cortex, thereby converting a 31-A2 type fracture to a 31-A3 type fracture [22]. IM nails provide lower angles, and in the present study, none had a cutout. The disadvantages of IM nailing include potentially increased blood loss and higher cost of implants compared to DHS, a comparatively cheaper device associated with lower bleeding risks. When checking the prices of implants produced by all major orthopedic implant suppliers in the UK, the average cost for a sliding hip screw was estimated at £252.51, for a short intramedullary nail was estimated at £760.08, and for a long intramedullary nail was estimated at £1,175.40.

The limitations of this study include its retrospective nature and the sample size. Suggestions for future research in the same field can include the evaluation of the operative time, which may give an indication of the difficulty encountered in fracture reduction and implant placement, assessment for the presence or degree of any associated hip arthritis that may adversely affect fracture reduction maneuvers intraoperatively, and evaluation of the effect of nonsurgical factors (i.e., American Society of Anesthesiologists (ASA) physical status classification, medical comorbidities, etc.).

## Conclusions

This study demonstrated a 3.7% (four cases) screw cutout occurrence following a fracture of the neck of the femur surgery. These included two cases of TAD of less than 25 mm. However, they all used a 135° DHS plate. Using 130° or 125° fixed-angle intramedullary devices allows a better position of the lag screw and minimizes the risk of screw cutout and other complications.
